# A Snapshot of CNVs in the Pig Genome

**DOI:** 10.1371/journal.pone.0003916

**Published:** 2008-12-16

**Authors:** João Fadista, Marianne Nygaard, Lars-Erik Holm, Bo Thomsen, Christian Bendixen

**Affiliations:** Group of Molecular Genetics and Systems Biology, Department of Genetics and Biotechnology, Faculty of Agricultural Sciences, Aarhus University, Tjele, Denmark; Max Planck Institute for Chemical Ecology, Germany

## Abstract

Recent studies of mammalian genomes have uncovered the extent of copy number variation (CNV) that contributes to phenotypic diversity, including health and disease status. Here we report a first account of CNVs in the pig genome covering part of the chromosomes 4, 7, 14, and 17 already sequenced and assembled. A custom tiling oligonucleotide array was used with a median probe spacing of 409 bp for screening 12 unrelated Duroc boars that are founders of a large family material. After a strict CNV calling pipeline, 37 copy number variable regions (CNVRs) across all four chromosomes were identified, with five CNVRs overlapping segmental duplications, three overlapping pig unigenes and one overlapping a RefSeq pig mRNA. This CNV snapshot analysis is the first of its kind in the porcine genome and constitutes the basis for a better understanding of porcine phenotypes and genotypes with the prospect of identifying important economic traits.

## Introduction

The pig (Sus scrofa) is a cetartiodactyl mammal from a different clade than rodents and primates and last shared a common ancestor with humans approx. 83 million years ago [1]. The porcine genome has an estimated size of 2.7 Gb, consisting of 18 autosomes and the X and Y sex chromosomes [2]. Genomic comparisons between the pig and human have unravelled more structural resemblance than, for example, mouse and human [2]–[4]. Pig is also a more trustworthy animal model for human disease since its physiological and anatomical resemblance is far greater than any other laboratory species. Consequently, the pig has been used progressively as a model within the human health research in e.g. obesity, cardiovascular disease, arthritis, diabetes, hypertension, cancer, organ transplantation, and Alzheimer's disease [5]–[8]. Further to the biomedical relevance, the pig is of great agricultural importance as the main source of animal protein world-wide (Porcine sequencing white paper).

Recently it has been reported that structural variation, like copy number variants (CNVs), is genome-wide present not only in humans [9]–[17] but also in chimpanzees [18]–[19], mice [20]–[23], nematodes [24], fruit fly [25], and cow [26]. CNVs represent segments of DNA larger than 1 kb present at a variable copy number in comparison with a reference genome [27] and they can be responsible for altered gene expression [28] leading to striking phenotypic variance including disease associated traits [29]–[30]. Despite numerous studies, no assessment of the extent and impact of CNVs in the pig genome has been made until now.

Based on a pig family material comprising 14 boars, 700 sows and about 12,000 offsprings this paper presents a preliminary analysis of CNVs detected in the genomes of twelve of the boar founders compared to one unrelated Hampshire boar, using high density tiling-path oligonucleotide array comparative genomic hybridization technology (array CGH) [31]. The designed arrays encompass part of the chromosomes 4, 7, 14, and 17 from the August 2007 preliminary assembly release with a median probe spacing of 409 bp. After a stringent pipeline, the analysis led to the identification of 37 copy number variable regions. Chosen CNVs were further confirmed by RT-PCR [32].

As the first of its kind in pig, this study examines the extent and pattern of CNVs in the pig genome, important for future studies associating phenotype to genome architecture.

## Results

### Study design

Array CGH was carried out using an array comprising 384,979 oligonucleotide probes covering the preliminary pig genome assembly for part of the chromosomes 4, 7, 14, and 17 with a median probe spacing of 409 bp. Copy number variation was assessed by equating the log2ratio of signal intensity between the reference and test samples. Given the relative type of these comparative data, it was not possible to unequivocally ascertain the real status of the CNVs not RT-PCR validated, and hence whether they were deletions or duplications in the reference or in the test samples. Therefore, the status of the copy number variations reported here is in relation to the reference sample.

**Figure 1:**
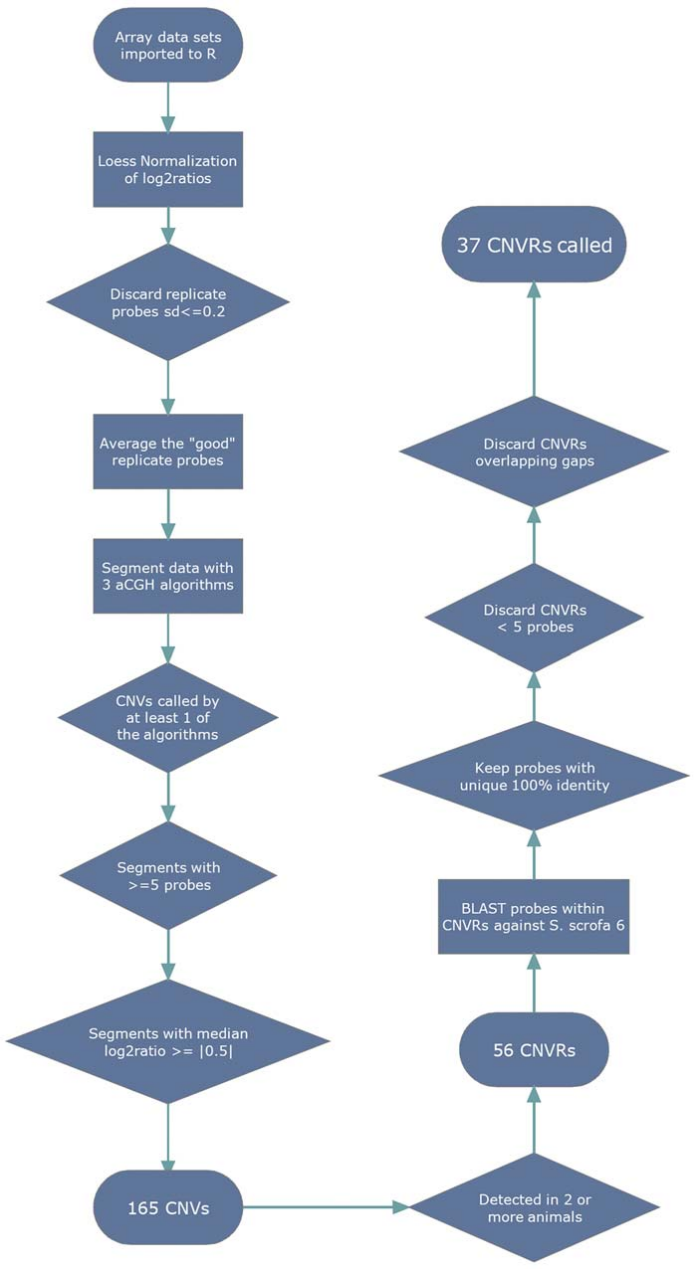
Methodological pipeline for assessing copy number variation in this study. See *Methods* section for detailed description.

Since our criteria of CNV detection (*Methods* and Figure 1) only permit to call a CNV if it is detected in at least two animals, we will be referring to copy number variable regions or CNVRs (merging of overlapping CNVs in two or more animals) instead of CNVs. The possibility that true CNVs exist within the loci discovered only in a single animal is acknowledged, since they may comprise sporadic cases, but in order to minimize the false positive rate, we focused only on CNVs found in two or more animals. Another strong reason to discard CNVs found in only one animal relates to the fact that some CNVs may be somatic and not germline [10], [60]–[61]. Therefore, in this study, as previously [10], a CNV was considered to be “germline” if it was detected in at least two animals.

### Pattern and frequency of CNVRs

**Figure 2:**
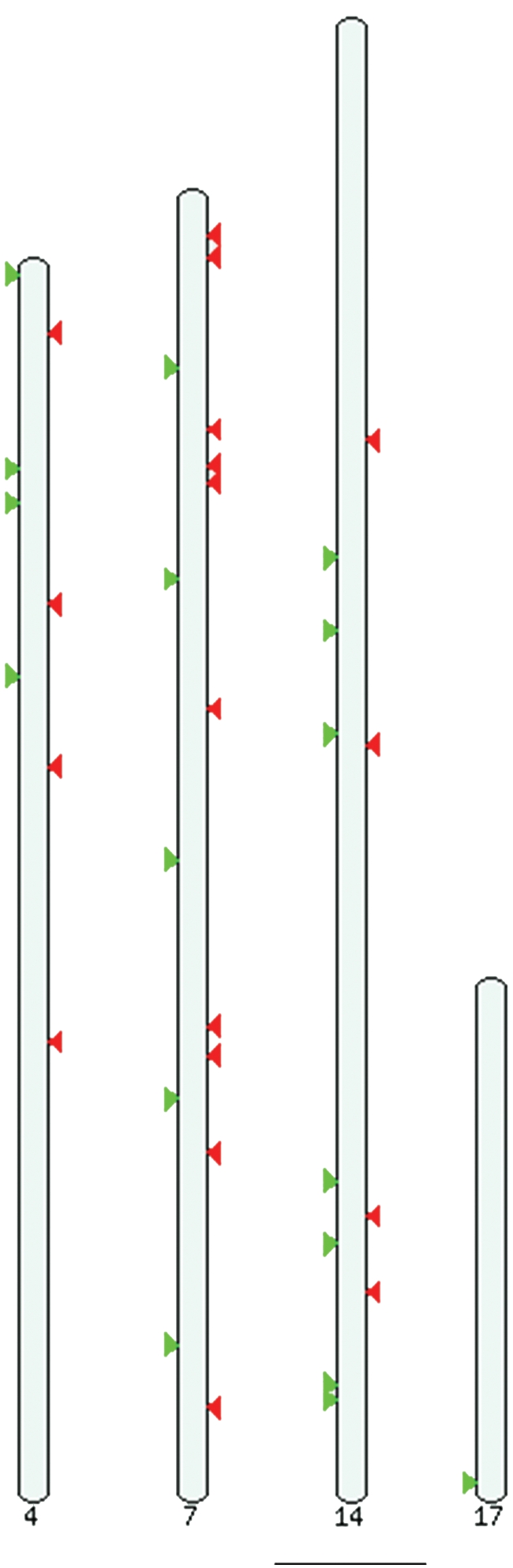
Ideogram from the *Sus scrofa* (May 2008 assembly) representing the CNVRs detected in our study. Losses are depicted in red while gains are in green. Not all the 37 CNVRs are visible since some are very close to each other.

Following the methodological copy number pipeline, 37 CNVRs were identified by array CGH across all the four chromosomes queried (Figure 2) with the proportion of any given chromosome amenable to CNVR varying from 0.03% to 0.31%. In summary, 19 (51.4%) CNVRs were called in two animals and the remaining (48.6%) called in three or more animals. Concerning copy number status, 18 (48.6%) were called as gains and the remaining 19 (51.4%) called as losses (Table 1).

**Table 1:** Distribution, length(bp), status and frequency of the 37 CNVRs detected by array CGH.

CNVR ID	Chr	Start	End	Length	Status	Animals	Pig Unigenes
1	4	1 695 691	1 703 117	7 427	Gain	2	
2	4	7 560 864	7 564 463	3 600	Loss	3	
3	4	21 108 665	21 114 596	5 932	Gain	2	
4	4	24 510 700	24 517 176	6 477	Gain	9	
5	4	34 556 025	34 562 828	6 804	Loss	3	
6	4	41 748 621	41 758 338	9 718	Gain	2	
7	4	50 753 651	50 761 743	8 093	Loss	4	
8	4	78 167 375	78 177 556	10 182	Loss	8	
9	7	4 502 382	4 510 505	8 124	Loss	2	Ssc.18508
10	7	6 630 532	6 636 941	6 410	Loss	2	
11	7	17 630 828	17 634 369	3 542	Gain	2	
12	7	23 821 562	23 838 973	17 412	Loss	10	
**13**	**7**	**27 171 334**	**27 203 171**	**31 838**	**Loss**	**4**	
14	7	27 660 543	27 699 166	38 624	Loss	3	
15	7	29 138 825	29 161 420	22 596	Loss	2	
**16**	**7**	**38 743 324**	**38 746 353**	**3 030**	**Gain**	4	
17	7	51 627 146	51 633 742	6 597	Loss	2	
18	7	66 692 031	66 753 950	61 920	Gain	10	
19	7	83 324 440	83 331 849	7 410	Loss	2	
**20**	**7**	**86 241 308**	**86 244 612**	**3 305**	**Loss**	3	
21	7	90 510 071	90 513 252	3 182	Gain	2	
22	7	95 943 885	95 949 065	5 181	Loss	2	
23	7	115 040 167	115 050 693	10 527	Gain	3	
**24**	**7**	**121 271 511**	**121 276 035**	**4 525**	**Loss**	2	
25	14	41 934 539	41 940 418	5 880	Loss	2	
26	14	53 639 912	53 651 468	11 557	Gain	3	
27	14	60 889 473	60 895 547	6 075	Gain	6	
28	14	71 252 362	71 281 840	29 479	Gain	6	
**29**	**14**	**72 268 017**	**72 274 799**	**6 783**	**Loss**	2	
30	14	115 689 469	115 733 497	44 029	Gain	4	Ssc.7991, Ssc.52309
31	14	119 256 322	119 263 779	7 458	Loss	2	
32	14	122 029 011	122 035 919	6 909	Gain	2	
33	14	126 779 081	126 784 999	5 919	Loss	7	
34	14	136 045 554	136 047 297	1 744	Gain	2	
35	14	137 517 567	137 521 449	3 883	Gain	2	
36	17	50 258 487	50 260 569	2 083	Gain	2	
37	17	50 554 656	50 559 679	5 024	Gain	3	

CNVRs indicated in bold overlap segmental duplications.

The genomic coordinates are relative to the Sus scrofa May 2008 assembly.

Previously, it has been suggested that deletions are under stronger purifying selection than duplications [33]. If so, deletions should be both less frequent and shorter than duplications. When comparing the length and number of gains versus losses in the CNVRs, practically the same number was detected (see above) while the total size of the gains was about 16 kb larger than the size of the losses (although according to the Wilcoxon rank sum test not statistically significant at p value ≤0.01).

**Figure 3:**
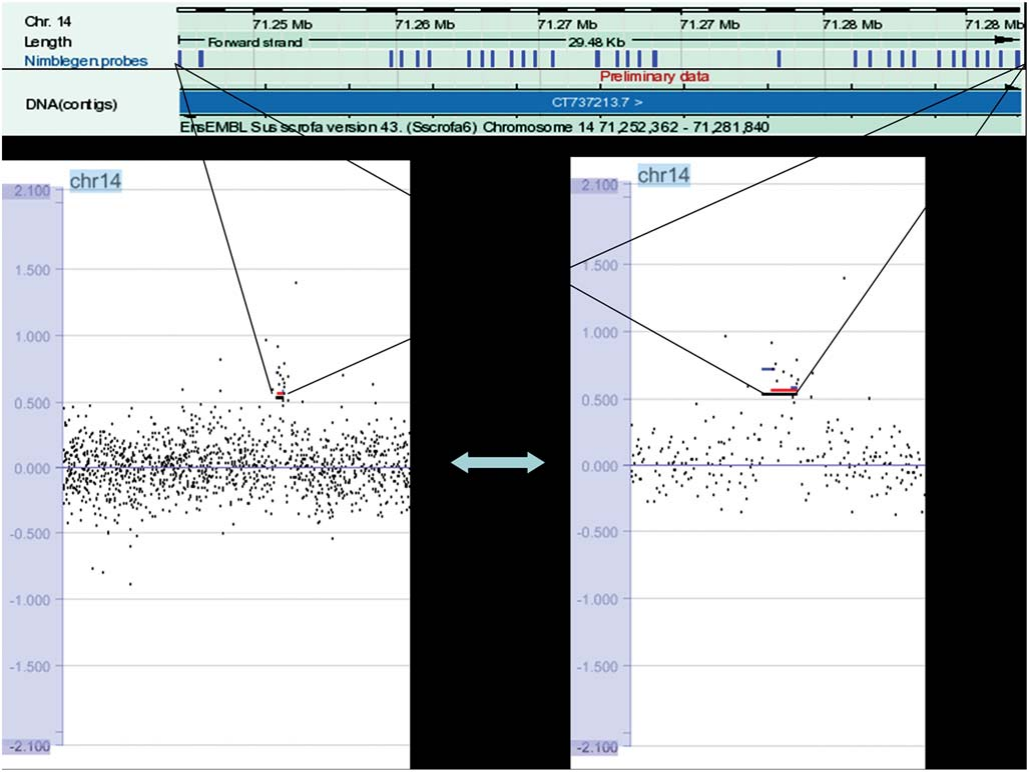
Example of a CNVR. CNVR 28 is shown (zoomed out on bottom left; zoomed in on bottom right) with log2ratio along with the probes (blue bars) covered in this region. Images from Nimblegen SignalMap™ and Ensembl [39].

Copy number regions were discarded if (1) they overlapped more than one contig and (2) contained gaps due to the high error rate of this preliminary assembly. This was done because contigs can be misassigned and gaps may contain future contigs that can result in the disruption of the CNVRs called (see Figure 3 for an example of a CNVR called). The CNVRs ranged in size from 1.74 kb to 61.92 kb with a mean of 9.32 kb and a median of 6.89 kb, covering 429.269 kb (0.18%) of the 237.76 Mb of sequence addressed (Table 2).

**Table 2:** Summary of the CNVR content (in bp) and sequence covered (including gaps) by the oligo array CGH probes.

Chr	CNVRs	Median size	Mean size	Size range	CNVR Content	Sequence covered	% CNVR
4	8	7 116	7 279	10 182–3 600	58 223	52983989	0.11
7	16	7 004	14 639	6 1920–3 030	234 223	76062953	0.31
14	11	6 783	11 792	44 029–1 744	129 716	83175859	0.156
17	2	3 554	3 554	5 024–2 083	7 107	25535213	0.03
All	37	6 894	9 316	61 920–1 744	429 269	237758014	0.18

When querying the part of the genome covered by CNVRs for the greatest divergence in genome size between two animals among our set, it was found that animal B had the biggest net gain, spanning 57.56 kb over five CNVRs, while animal E had the biggest net loss with −106.6 kb over eleven CNVRs. Comparison of these genomes disclosed a difference of 164.1 kb in size between these two animals.

There are seven CNVRs that are apparently aberrant in the genomes of at least half of the test boars. However, these CNVRs are most likely to be aberrant regions in the reference boar since this is of a different breed (i.e. Hampshire), and probably more structural genomic variation is present between breeds than within the same breed, since all the test boars are of the Duroc breed. This hypothesis remains to be tested.

Previous analyses have reported enrichment of CNVs near segmental duplications (sequences ≥1 kb in size with sequence identity ≥90% [34]) in humans [9]–[12], [16]–[17], mice [20]–[23], and chimpanzees [19]. Segmental duplications have been mapped in the genomes of human [35], chimpanzee [36], and mouse [37], but the incomplete and highly error-prone preliminary assembly of the pig genome prevents us from drawing such a map. Consequently, only the available numbers can be focused on in this study. Among the regions found to contain CNVs, five (13.5%) overlapped segmental duplications (Table 1 and *Methods*).

Despite the discovery of CNVRs overlapping segmental duplications, it is important to note that our array probe design is biased against the detection of CNVRs that coincide with sites of segmental duplication because it only allowed probes that had a unique match in the genome (*Methods*).

### Functional analysis

In order to assess the gene content within the CNVRs reported, a sequence similarity ≥98% search between the pig Unigene database and the CNVRs was made and three pig Unigenes were retrieved (Table 1). Since a CNV can also affect gene expression at long distances [28] an additional search was performed for pig Unigenes that showed ≥98% sequence similarity with the contigs where the CNVRs are. Contigs with CNVRs range in length from 5.577 kb from 245.924 kb. Further there were nine new Unigenes in eight of the contigs (*CNVR.1* - Ssc.28459; *CNVR.11* - Ssc.25025; CNVR.12 - Ssc.26197; *CNVR.13* - Ssc.26126; *CNVR.14* - Ssc.42797, *CNVR.25* - Ssc.8364; *CNVR.31* - Ssc.38482, Ssc.14020; *CNVR.33* - Ssc.63374) .

Searching the Refseq mRNA database for vertebrate mammals, a gene was identified (95% sequence id) as part of the contig containing the CNVR 33. This Refseq mRNA corresponds to the ADRA2 gene, encoding the alpha2A-adrenergic receptor - a transmembrane receptor belonging to the rhodopsin family from which genes have been consistently reported to overlap CNV regions in other mammals [16], [19], [21]–[23]. In fact, this gene is actually overlapped by a putative human CNV, as seen in the Database of Genomic Variants [9].

### Validation by RT-PCR

**Figure 4:**
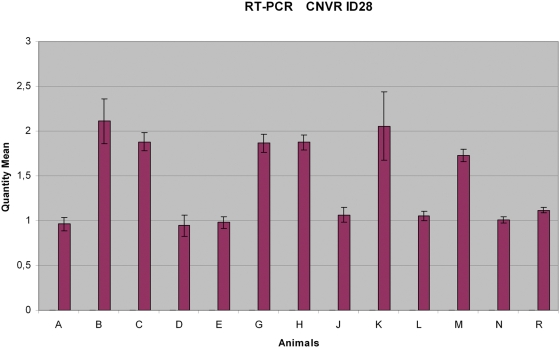
RT-PCR for the CNVR ID 28. A gain is shown in 6 of the test animals relative to the reference (R), as predicted by the pipeline.

Validation of the results was made with RT-PCR [32] on eight genomic regions (Figure 4) selected to represent a range of amplifications and deletions (CNVR IDs 2, 6, 7, 12, 23, 28, 33, and 37). The Data S1 file contains primer sequences, RT-PCR results, and the correlation between the array CGH and RT-PCR.

From these eight regions, four were confirmed (CNVR IDs 2, 7, 28, and 37). For CNVR 2, an additional animal having this CNVR was found by RT-PCR which was not expected from the array data. Regarding the CNVR 7, a loss was found not only in the four animals predicted by our CNV calling pipeline but also in all the other test animals relative to the reference. For CNVR 37, the PCR was negative in the reference animal, while the test animals gave well-shaped sigmoidal curves, suggesting a loss in the reference animal (data not shown). Three regions were not confirmed (CNVR IDs 12, 33, and 23) and one gave ambiguous results (CNVR ID 6).

## Discussion

Here, using a custom tiling oligonucleotide array CGH approach, we reported the first CNV survey of the pig genome among twelve unrelated healthy boars which are founders of a vast pig family. It should be stressed that only four chromosomes and not the whole genome were screened here. Both gains and losses of different lengths were discovered on part of chromosomes 4, 7, 14, and 17. With the tiling nature of the array, we were able to identify 37 frequently occurring loci of copy number variation.

Natural large-scale genomic size divergence between animals of the same breed was found to vary by at least 164.1 kb, showing that a substantial portion of the pig genome may vary in copy number. In comparison with CNV studies in the “finished” human and mouse genomes [17], [22], our study found an order of magnitude less genomic size divergency. This is not surprising since the pig assembly is currently only in its draft form, covering less sequenced data.

With a detection sensitivity ranging from about 2 kb (median spacing*5probes) to 248.471 kb (length of the biggest contig in the Sscrofa 6 assembly), at least 0.18% of the mapped pig chromosomes are tolerant to copy number variation.

Concerning the functional sequence content, twelve pig unigene sequences and one Refseq gene were found to be putatively under influence of the CNVRs. The Refseq gene is related to sensory perception, which is a common large and rapidly evolving gene family found to contain many genes overlapped by CNVs in other mammalian genomes [16]–[17], [19], [21]–[22]. This gene family is possibly conserved by natural selection in mammalian species or, with a different view, could mean a relative relaxation of selective pressure on copy number variants for these genes.

In order to confirm the CNVRs found with the array approach, RT-PCR was carried out on some CNVRs and 50% of the selected CNVRs were validated. Although this validation rate seems poor, it should be noted that RT-PCR is not trivial for a highly error-prone preliminary genome assembly. Many factors could account for this discrepancy as explained very thoroughly elsewhere [63], like: (1) The breakpoint estimation of the copy number variable regions may not be correct leading to a primer design upstream or downstream of the true boundaries of the CNVR; (2) CNVRs have a lower probe density than usual because some regions surrounding the Nimblegen probes have a high repeat content which may disturb the PCR reaction; (3) The animals may have SNPs and small indels in the CNVRs compared to the reference genome assembly, which may compromise the RT-PCR reaction but not the CGH hybridization, or at least not so seriously [62], since the RT-PCR primers are shorter and thus less robust than the CGH probes. The source of the disagreement between RT-PCR and array CGH awaits further research.

Further validation was done using 7k SNPs ascertained in-house by mapping their surrounding sequences to the *Sus scrofa* 6 assembly for the pigs queried in this study (unpublished data). Here it was tested whether the SNPs found in close proximity of the CNVRs validated by RT-PCR gave some information about the presence of copy number variants in those regions (see *Methods*). In fact, for three of the CNVRs, the SNP alleles in close proximity were found to cluster in 2 groups: animals with the CNVR had one set of alleles while the others had a different set of alleles. Probably due to the low density distribution of SNPs they were uninformative regarding the status of the other putative copy number variable regions.

Since our analytical pipeline for measuring the pig CNV landscape was developed in order to minimize the detection of somatic CNVs and false positives, and since the pig preliminary assembly contains high amounts of unfinished sequence and incorrectly mapped regions, our results are an obvious underestimate of the total number of CNVs in the sequences covered. As an example, when allowing copy number variants to be called in only one animal, there is an increase in the CNVR estimate from 37 to 165 (unpublished data).

It is also important to state that the sequences within a contig might be incorrectly assembled. Consequently, a CNVR detected at a certain position and in a certain orientation within a contig might have a different position and orientation within this contig. This could affect the performance of the calling algorithms. Future pig genome assemblies will shed light on this matter.

With the hypothesis that hundreds or maybe thousands of CNVs exist in the pig genome, this study is still an early step toward a more complete understanding of copy number variation within the pig species. Consequently, more studies are needed to fully understand the extent and functional roles of CNVs. Therefore, integration of previously gathered QTL and SNP (unpublished data) data for the pig families, the CNV data reported here, and a more comprehensive genome-wide CNV study in our group will certainly provide a framework for genetic association studies that will hopefully unravel the biological relevance of genetic variation and their effect upon important economic traits.

## Methods

### Oligonucleotide array CGH

A custom 385k tiling-path array CGH was designed (Nimblegen Systems, http://www.nimblegen.com) to cover the preliminary *Sus Scrofa* assembly for chromosomes 4, 7, 14, and 17, from the August 2007 release (http://www.sanger.ac.uk/Projects/S_scrofa/), which was the newest release at the time of the experiment. The tiling-path array covers the chromosome sequences of the August 2007 release up to the old chromosome endpoint coordinates of the previous release (April 2007).

The probe design fundamentals are described in the Nimblegen technical note http://www.nimblegen.com/products/lit/probe_design_2007_11_13.pdf). Briefly, highly repeated elements in the genome were repeat-masked with a strategy similar to the WindowMasker program [40]. Concerning uniqueness, probes having a unique genome sequence match were selected with SSAHA [41]. An isothermal format (Tm  =  76°C) [42] and probe length constraint between 50 and 75 bp were used for probe synthesis.

The probes were integrated into an array design using ArrayScribe™, which resulted in a design with a median probe spacing of 409 bp. The arrays were manufactured by maskless array synthesis technology and the oligonucleotides were synthesized on the arrays by photolithography [43]–[44].

### Sample preparation

From a pig family-material comprising 14 boar founders, 700 sows, and about 12.000 offspring, 12 of the Duroc boar founders (A, B, C, D, E, G, H, J, K, L, M, and N) were selected to function as test animals. An unrelated Hampshire boar was selected as the common reference. We adhered to our institutional guidelines for the ethical use and treatment of animals in experiments.

Genomic DNA from boar N and the reference animal was isolated from lung/liver tissue by the use of Genomic-tip 100/G, Genomic DNA Buffer Set and Genomic DNA Handbook from Qiagen. After precipitation, the isolated DNA was resuspended in 1×TE-buffer (10 mM Tris-HCl, 1 mM EDTA; pH 8).

For the first 11 of the 12 test boars, genomic DNA was purified from blood to a concentration of ∼250 ng/µL by salt precipitation. The precipitation was performed by adding 2.3×volumes of cold 96% ethanol and 0.1×volumes of 3M sodium acetate (pH 4.8). After 20 minutes of centrifugation at 4°C and 13.000 rpm, the precipitated DNA was washed with 70% ethanol, re-precipitated by 5 minutes of centrifugation at 4°C and 13.000 rpm. After precipitation, the isolated DNA was resuspended in 1×TE-buffer to a concentration of ∼250 ng/µL.

Following the isolation of the 13 genomic DNA samples, the DNA quality was assessed by measuring the concentration and the purity on the NanoDrop ND-1000 Spectrophotometer (NanoDrop Technologies). DNA integrity and purity were also assessed by gel electrophoresis on a 1% agarose gel (SeaKem® GTG® Agarose, Cambrex Bio Science, and 1×TBE buffer, Invitrogen) containing 0.15 µg/mL ethidium bromide.

DNA fragmentation, labelling, and hybridization were carried out according to the manufacturer's protocol. Briefly, the DNA was fragmented by sonication and labelled with Cy3- and Cy5-labelled 9mer primers. Each of the 12 boars was hybridized twice (technical replicates, 24 arrays) against the common reference with a MAUI hybridization system (BioMicro Systems). Scanning and intensity feature extraction were made as previous [45].

### Statistical analysis

The array data sets were imported into the R statistical programming language version 2.6.0 [46]. The intensity log_2_ ratios of the test versus reference samples were normalized with the Loess function from the Bioconductor [47] limma package [48].

In order to decrease the background noise of the arrays and retrieve only high-confident probes for each pair of technical replicate arrays, the probes that didn't have a standard deviation of log2 ratio ≤0.2 were discarded while the others were averaged. The averaged normalized log2 ratios were used as input for the Bioconductor package snapCGH [49]. Segmentation of the data was then performed with three algorithms available in this package, DNAcopy [50], GLAD [51] and HomHMM [52]. The reason for using all these three algorithms instead of only one is that each one has a different approach to the segmentation scheme, providing both advantages and disadvantages in CNV detection [53]–[54]. Consequently, when using all these algorithms the false negative rate of CNVs detection is decreased, while the possible increase in false positives is addressed by using the following downstream filter criteria.

CNVRs were called as the segments found by at least one of the mentioned algorithms with ≥5 consecutive probes, a median log2 ratio of ±0.5 and detected in two or more animals.

Subsequently, we used Tera-Probe™ [55] an algorithm similar to the BLAST sequence search algorithm [56] but optimized for small oligonucleotide sequences. Tera-Probe™ was used to query the probes within the CNVRs against the newest available version of the *Sus Scrofa* assembly (*Sus Scrofa* 6, May 2008 release), and probes were only kept if they had a unique optimal hit (100% sequence identity). Concerning this filtering criterion, we also kept the probes if there were two or more regions in the assembly that had a perfect hit from a block of at least half of the probes from a CNVR, since it was evident that these probes queried a putative segmental duplication. The CNVRs that decreased the number of probes to less than 5 were discarded. Finally, the remaining CNVRs were retained if they did not overlap any gap in the *Sus scrofa* 6, May 2008 release assembly.

The NCBI's pig Unigene database release 34 [57], based mainly on the data generated by the Sino-Danish Pig Genome Sequencing Project [58], the Refseq vertebrate mammalian mRNA database release 27 [57], and the *Sus scrofa* version 6, May 2008 release (http://www.sanger.ac.uk/Projects/S_scrofa/) was implemented to run on a DeCypher computer (http://www.timelogic.com).

The Tera-BLAST™N sequence similarity algorithm was used to query the CNVR sequences against the pig Unigene and the Refseq vertebrate mammalian mRNA databases. Hits were retained if they had an E-value ≤1e-15 and if their sequence aligned ≥95% (from Refseq) and ≥98% (from Unigene) with a CNVR.

About 7k ∼120 bp sequences around SNPs ascertained in-house (unpublished data) from the animals queried in the CNV study were also queried against the *Sus scrofa* version 6, May 2008 release with an E-value ≤1e-15 and they were retained if they had a perfect hit in the chromosomes 4, 7, 14 and 17.

In order to check if the CNVRs overlapped any segmental duplication, Tera-BLAST™N was used to query the CNVRs sequences, which are all above 1 kb in size, against the *Sus Scrofa* version 6, May 2008. Sequences were retained if they had > = 1 kb and > = 90% identity.

The full data set from the oligo array CGH experiments has been submitted to GEO [59] under the accession ID GSE10753.

### Quantitative Real Time PCR

Determination of copy number variation by quantitative real time PCR was performed using the Applied Biosystems 7900HT Sequence Detection System and analyzed with the SDS 2.2 software following the guidelines of the manufacturer (Applied Biosystems). The primers and probes (Universal ProbeLibrary Probes, Roche Applied Science) were designed using the ProbeFinder software from Roche Applied Science (https://www.roche-applied-science.com/sis/rtpcr/upl/acenter.jsp?id030000) and are available in the supplementary data file. A serial dilution of genomic DNA from the common reference animal was used as template for creating a standard curve for each primer pair. The copy number of each CNVR was normalized against a control region in the genome that does not vary in copy number between the pigs. All PCRs (10 µL) were run in triplicate in 1× TaqMan Universal PCR Master Mix, 100 nM of each primer, 250 nM probe and 10 ng of genomic DNA. PCRs were run as follows: 10 min at 95°C followed by 40 cycles at 95°C for 15 sec and 58°C for 10 sec.

## Supporting Information

Data S1 RT-PCR primers, results and correlation with array CGH
(0.07 MB XLS)
